# Osteopontin and phospho‐SMAD2/3 are associated with calcification of vessels in D‐CAA, an hereditary cerebral amyloid angiopathy

**DOI:** 10.1111/bpa.12721

**Published:** 2019-04-04

**Authors:** Laure Grand Moursel, Linda M. van der Graaf, Marjolein Bulk, Willeke M.C. van Roon‐Mom, Louise van der Weerd

**Affiliations:** ^1^ Department of Human Genetics Leiden University Medical Center Leiden the Netherlands; ^2^ Department of Radiology Leiden University Medical Center Leiden the Netherlands

**Keywords:** cerebral amyloid angiopathy, collagen 1, hereditary cerebral hemorrhage with amyloidosis‐Dutch type, osteopontin, TGFβ, vascular calcification

## Abstract

In severe forms of cerebral amyloid angiopathy (CAA) pathology, vascular calcification has been observed in the cerebral cortex, both *in vivo* on MRI and CT, and post‐mortem using histopathology. However, the pathomechanisms leading to calcification of CAA‐laden arteries are unknown. Therefore, we investigated the correlation between calcification of cortical arterioles and several potential modulators of vascular calcification using immunohistochemistry in a unique collection of brain material of patients with a hereditary form of CAA, namely hereditary cerebral hemorrhage with amyloidosis‐Dutch type (HCHWA‐D or D‐CAA).

We show a topographical association of osteopontin (OPN) and TGFβ signaling factor phospho‐SMAD2/3 (pSMAD2/3) in calcified CAA vessel walls. OPN and pSMAD2/3 gradually accumulate in vessels prior to calcification. Moreover, we found that the vascular accumulation of Collagen 1 (Col1), OPN and pSMAD2/3 immunomarkers correlated with the CAA severity. This was independently of the vessel size, including capillaries in the most severe cases. We propose that calcification of CAA vessels in the observed HCHWA‐D cases may be induced by extracellular OPN trapped in the fibrotic Col1 vessel wall, independently of the presence of vascular amyloid.

## Introduction

Hereditary cerebral hemorrhage with amyloidosis‐Dutch type (HCHWA‐D) is an autosomal dominant genetic disorder caused by a missense mutation on chromosome 21 in the amyloid precursor protein (APP, NP_000475.1:p.Glu693Gln, known as APP E693Q) 29. This so‐called Dutch variant or D‐CAA, is characterized by early onset cerebral amyloid angiopathy (CAA) defined by a progressive accumulation of Amyloid beta (Aβ) in the cerebral vasculature, resulting in acellular thickening of the vessel wall 1, leading to recurrent hemorrhagic strokes with midlife onset 15, 18. CAA pathology is also present in the majority of Alzheimer’s Disease (AD) patients and is associated with intracerebral hemorrhages in the elderly.

Recently, a striped pattern of hypointense lines perpendicular to the pial surface was observed on *in vivo* 7T MRI in the occipital cortex of symptomatic D‐CAA patients 17. Histopathological examination showed that the striped pattern in the cortex on 7T MRI is caused by iron accumulation and calcification of penetrating arterioles 5, 39.

Similar calcifications have been observed in other hereditary CAA variants, such as the Iowa and Italian variants 26, 33, 44. In sporadic CAA, calcification of cortical areas involving CAA‐affected vessels seems to be much rarer than in the familial cases; its has only been described in three cases of CAA‐associated cerebral hemorrhage 5, 20, 28. Intracranial vascular calcifications in general aging and in neurodegenerative diseases are frequently found in the basal ganglia 23, 41, but cortical calcification has not been reported. Therefore, calcifications of the CAA‐vessels are thought to be one of the CAA‐associated microvasculopathies, secondary to CAA 22, 39. However, it is unknown why some CAA‐vessels exhibit calcifications, whereas other vessels with similar CAA load do not.

In the current study, we investigated if vascular calcification in HCHWA‐D is linked to expression of several factors known to promote calcification, independent of CAA itself, namely transforming growth factor β (TGFβ), osteopontin (OPN) and collagen1 (Col1), as detailed below. We previously reported an upregulation of the TGFβ pathway in HCHWA‐D post‐mortem brain tissue 9, 10. In particular, we described in angiopathic vessels a correlation between CAA severity and the accumulation of phosphoSMAD2/3 (pSMAD2/3), a direct TGFβ1 downstream signaling factor 9. Since, it is known that TGFβ is a key factor in peripheral vascular calcifications 13, 40, we investigated the correlation between TGFβ regulation and calcification in individual vessels.

A second known modulator of vascular calcification is OPN, also known as secreted phosphoprotein 1 (SPP1). It is a non‐collagenous bone matrix protein induced by TGFβ, leading to vessel calcification in a wide variety of peripheral disorders, such as atherosclerosis, diabetes mellitus and chronic renal failure 14. In the central nervous system, OPN is a constituent of the normal extracellular matrix (ECM), but in the context of neurodegenerative diseases, OPN has been described as a cytokine with a dual role in neuroinflammation and neuroprotection 4, 6, 43. Very little is known about the role of OPN in cerebrovascular calcification, though one previous study showed OPN associated with calcifications in the basal ganglia of AD patients 8.

Finally, ECM‐related pathways are known to be upregulated in D‐CAA 10. In particular Col1, which is a pro‐fibrotic gene induced by TGFβ1 signaling and an OPN binder, was found upregulated in D‐CAA 9 and in an earlier study significantly increased quantities of Col1 were associated with vascular amyloid deposition 38. In atherosclerosis, Col1 is involved in vascular calcification through the binding of matrix vesicles that act as nucleation foci for calcium deposits 11.

In order to unravel these pathomechanistic factors associated with cortical calcification, we used post‐mortem human brain material of D‐CAA patients with well‐characterized different levels of CAA load 9. We investigated the different layers (luminal, medial and abluminal) of calcified vessels with standard immunomarkers for these vascular components and assessed in cortical area the association of pSMAD2/3, Col1 and OPN with calcification and disease severity.

## Material and Methods

### Brain tissue

Occipital post‐mortem brain tissue from D‐CAA patients was obtained from the Netherlands Brain Bank (NBB) and from our local neuropathology tissue collection (LUMC). Written informed consent was obtained for each donor and all material and data were handled in a coded fashion maintaining patient anonymity according to Dutch national ethical guidelines (Code for Proper Secondary Use of Human Tissue, Dutch Federation of Medical Scientific Societies, Rotterdam, the Netherlands). The study was approved by the local Ethics Committee.

### Experimental design

Brain tissue from eight D‐CAA patients, genetically tested for the presence of the mutation (NM_000484.3(APP):c.2077G>C), was used in this study as summarized in Table 1. In addition to the two cases previously described for the presence of calcifications (in 5; cases H2 and H14), six additional patients presenting a known range of CAA severity (based on 9) were selected (H5, H6, H7, H8, H9 and H10 as shown in Table 1).

**Table 1:** Demographics of cases, patient code and CAA severity based on 9

Diagnosis	Source	Age	Gender	PMD*	Code†	CAA severity‡
HCHWA‐D	LUMC	70	F	5	*H14* §	n.a.
HCHWA‐D	LUMC	57	M	3	H2§	+
HCHWA‐D	LUMC	50	M	19	H5	+
HCHWA‐D	LUMC	51	M	3	H8	+
HCHWA‐D	NBB	61	M	7	H6	++
HCHWA‐D	LUMC	71	M	n.a.	H10	++
HCHWA‐D	LUMC	67	F	n.a.	H9	+++
HCHWA‐D	NBB	71	M	6	H7	+++

Abbreviations: HCHWA‐D = hereditary cerebral hemorrhage with amyloidosis‐Dutch type; NBB = Netherlands Brain Bank; LUMC = Leiden University Medical Center; n.a. = not available.

* Post‐mortem delay (in hours).

† Patient code used in 9, (*added material in italic*).

‡ +: moderate, ++: high, +++: very high.

§ Patient material used in studies described in 5.

### Tissue sectioning and staining

Formalin‐fixed, paraffin‐embedded blocks of brain tissue were cut into serial 5 µm thick sections and mounted on coated glass slides (SuperFrost® Plus, VWR, Dietikon, Switzerland). Deparaffinization in xylene and rehydration through a series of ethanol concentrations was done. To detect calcification standard hematoxylin & eosin (HE) staining and the Von Kossa technique were included. For immunohistochemistry, the usage of an antigen retrieval procedure, a list of antibodies and their dilution are mentioned in Supporting Information Table S1. After blocking steps for endogenous peroxidase (3% H_2_0_2_ in dH_2_0 for 10 minutes) and for unspecific epitopes binding in blocking buffer (1% BSA suspension in 0.1% Tween 20, Phosphate Buffer Saline pH 7.4 for 1 h at room temperature), the sections were incubated with primary antibody overnight at 4°C in the blocking buffer. Incubation with secondary HRP antibody was followed by a DAB reaction (standard or enhanced using an avidin‐biotin complex; details in Supporting Information Table S1) and mounted with Entellan® New (107961, Merck, Darmstadt, Germany). An overview of the serial sectioning with the sequence of stainings is given in Supporting Information Table S2. All slides were digitized using an automatic bright field microscope (Philips Ultra Fast Scanner 1.6 RA, Philips, the Netherlands). All stained sections were independently evaluated by LvdG and LGM for the amount of different immunomarkers as described below.

### Quantification of severity grade of calcification, Col1 load, CAA load (Aβ), osteopontin load and pSMAD2/3 load

On the HE stained sections, three cortical areas were delimited at low magnification (2.016 mm^2^ per field of view), based on the presence of calcifications (deep purple color; 24). The corresponding area on consecutive slides immunostained for OPN, pSMAD2/3, Aβ and Col1 was identified for each patient. Every area was counted twice at higher magnification (6.897 mm^2^ per field view): one time for the total number of stained vessels and one time for the stained capillaries only. Subtraction of capillary number to total number of vessels gave the amount of intermediary size vessels. This group include all vessels larger than capillaries, with a typical external diameter between 10 and 300 μm. Leptomeningeal vessels were excluded. Of note, only fully calcified vessels with a deep purple color in HE were counted.

Statistical analysis was done using GraphPad Prism v7.00 with a level of statistical significance set at *P *< 0.05. Normality of the distribution was assessed with a D’Agostino & Pearson test. Correlation matrices were computed with a two‐tailed *P*‐value using Pearson correlation or with Spearman correlation in case of non‐parametric distribution.

### Quantification of immunomarkers in calcified vessel

Classic vascular immunomarkers were investigated in the calcified vessel layers (luminal, medial and abluminal). Vascular smooth muscle cells (VSMCs) are labeled with Smooth Muscle Actin (SMA) and the endothelial cells (ECs) with CD31, CD105, vWF and/or ICAM‐1. The collagen composition of the ECM was determined by Col4 and Col1.

Intact calcified vessels that could be identified on consecutive sections were scored for SMA, CD31, ICAM‐1, CD105, vWF, Col4 and Col1 in the luminal, medial and abluminal layers (examples are given in Supporting Information Figure S1). A maximum of 10 vessels per patient was graded, given a total of 48 vessels scored in the different patients: 0 vessel for H5; 3 vessels for H8; 10 vessels for H10; 4 vessels for H2; 3 vessels for H9; 10 vessels for H6, 8 vessels for H14 and 10 vessels for H7.

## Results

### Calcified vessels and localization of immunomarkers

We expect the majority of stained vessels to be small arteries and arterioles (primarily affected in this disorder), but a specific distinction with veins and venules was not assessed; therefore we use the term of “medium to large vessels.” Furthermore, we will employ the term calcification, more widely used than mineralization, with the understanding that the deposits may consist apart from calcium of several other co‐aggregating minerals, such as iron.

#### Calcification severity and occurrence of OPN and pSMAD2/3 labeling

**Figure 1:**
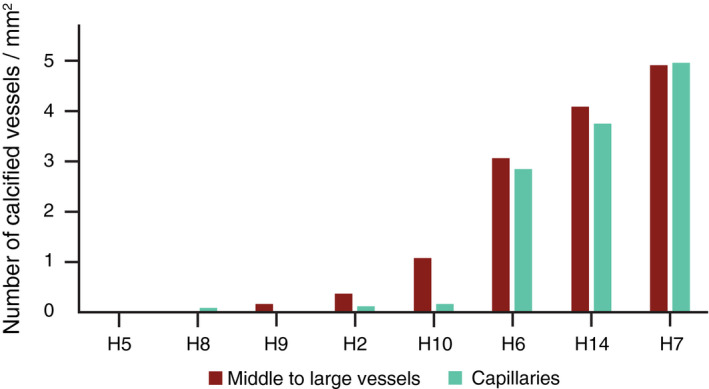
Calcification load (number of fully calcified vessels/mm^2^) in selected HCHWA‐D material ranked from left to right on increased calcified vessel load. Calcification load range from: absent (in H5); low (in H8); mild (inH9, H2 and H10) to heavy (in H6, H14 and H7).

Calcifications of angiopathic vessels were found in the occipital cortex of 7 out of 8 D‐CAA cases. We observed different calcified vessel load between the D‐CAA cases, expressed as the number of fully calcified vessels per mm^2^, as shown in Figure 1. Within individuals, calcified vessels were clustering in specific part of the tissue section, and confined to the middle cortical layers (as also described in 5). Calcified capillaries were Aβ‐laden capillaries (capCAA), restricted to areas where bigger calcified vessels were present (data not shown) and only observed in those cases with the most severe calcification load in the medium to large vessels (Figure 1).

**Figure 2:**
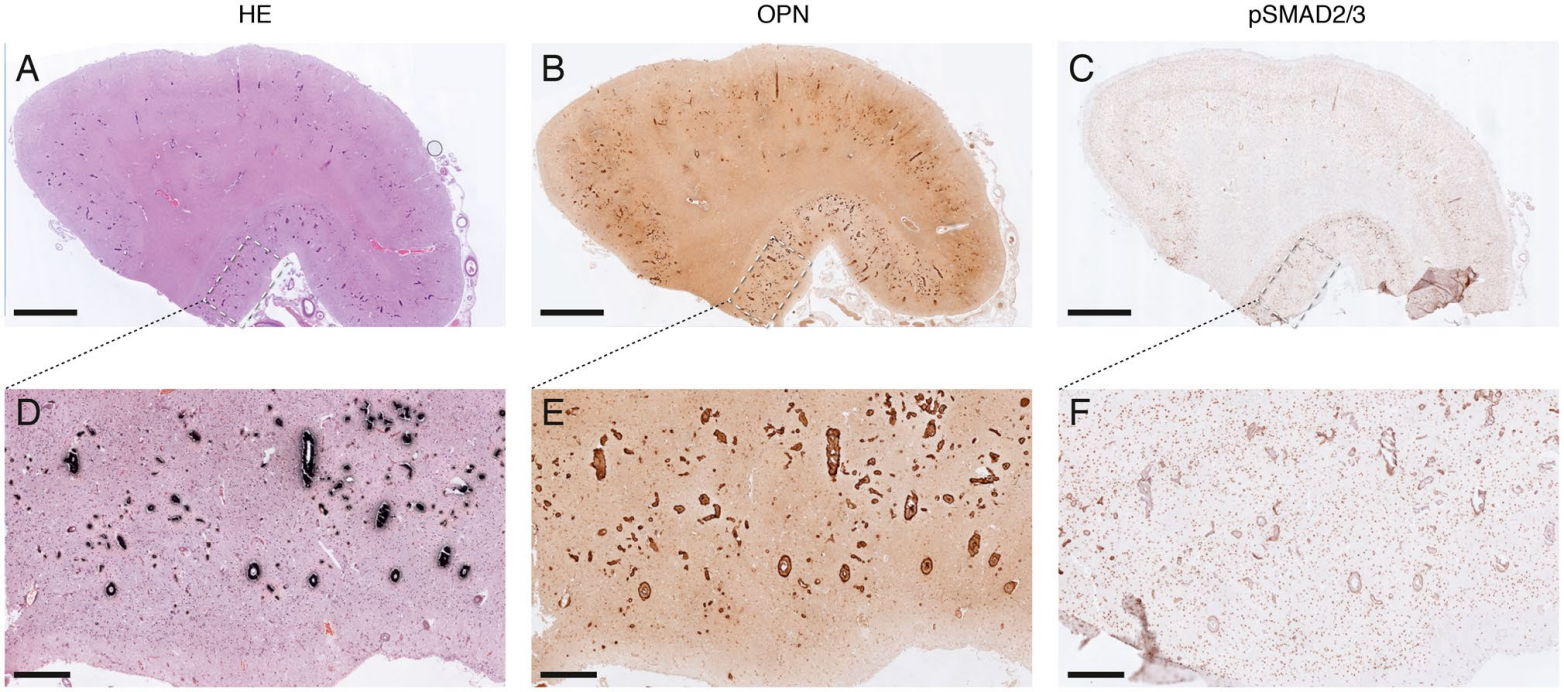
Topographical co‐localization of OPN and pSMAD2/3 staining in calcified vessel already detectable at low and medium magnification; H7 patient. A, D. von Kossa staining. B, E. OPN staining. C, F. pSMAD2/3 staining. (**A–C**) Overview, scale bar 2 mm; (**D–F**) Details of respectively (A–C); scale bar 400 μm.

**Figure 3:**
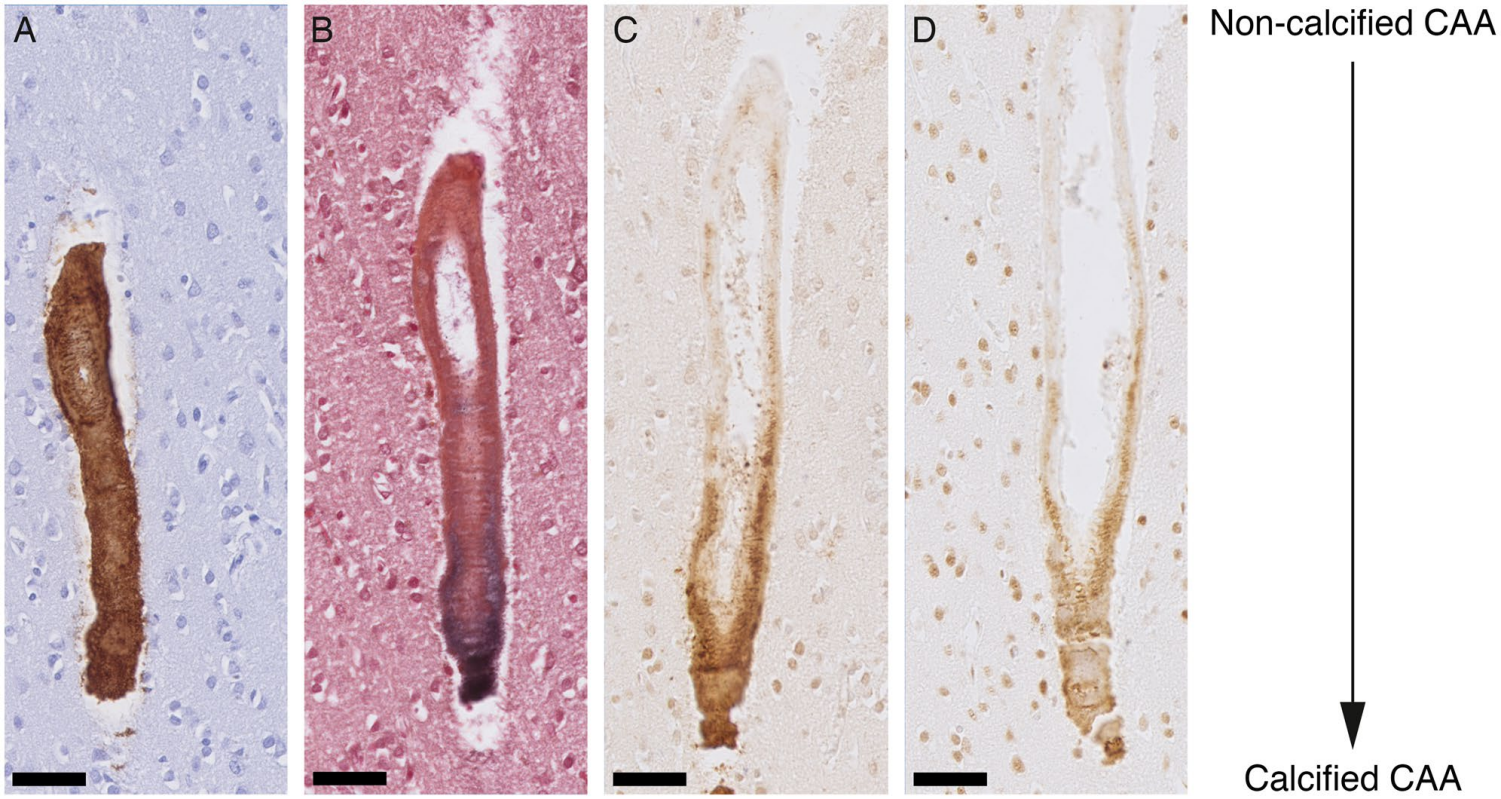
Transition CAA non‐calcified to calcified vessel showing the gradual medial OPN and pSMAD2/3 accumulation. Longitudinal sectioning of a penetrating artery oriented leptomeninges (top) to white matter (bottom) side; H2 patient. **A**. Aβ staining. **B**. von Kossa staining. **C**. OPN staining. **D**. pSMAD2/3 staining. AD. Consecutive slides; scale bar 50 μm.

**Figure 4:**
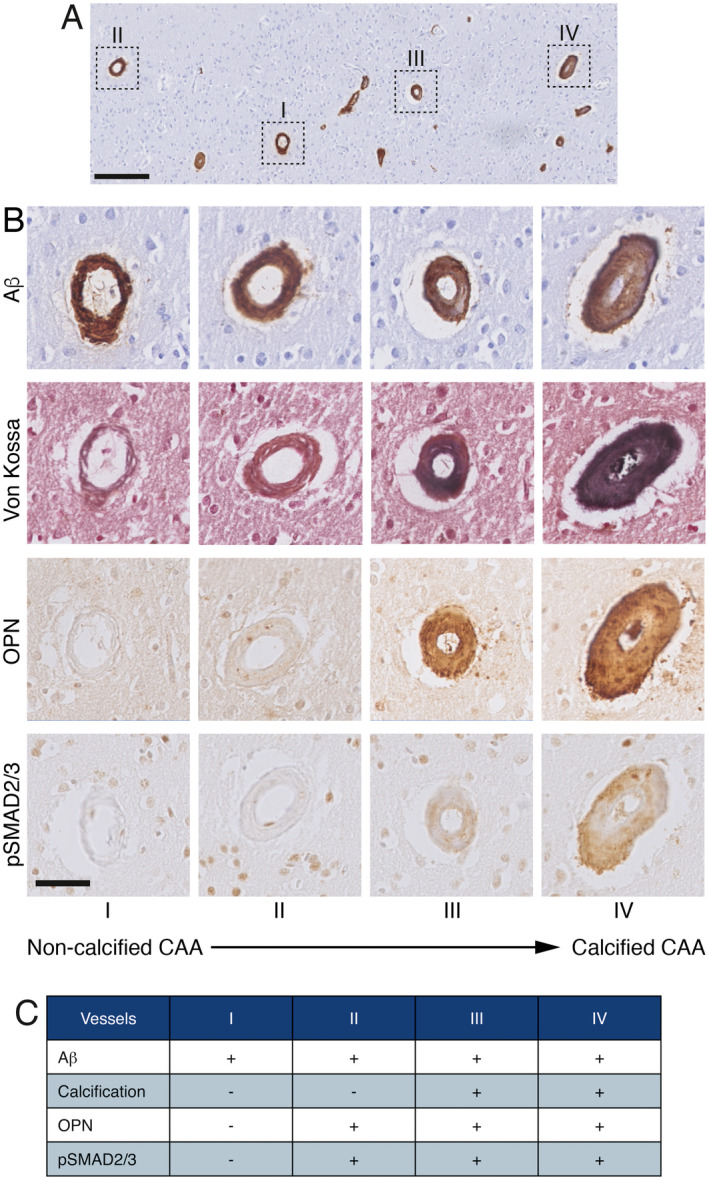
Adjacent cortical angiopathic vessels showing different degree of calcification and gradual accumulation of OPN and pSMAD2/3 in transverse sections, H2 patient. **A**. cortical area overview stained for Aβ; scale bar 200 μm. **B**. details of vessels I to IV identified in (A) and consecutive slides labeling, scale bar 50 μm. C. Table indicating the presence (+) or absence (‐) of labeling on the vessel wall.

Calcified vessels showed a topographical co‐localization with OPN and pSMAD2/3 staining in consecutive slides (Figure 2). Calcified vessels were always positive for both OPN and pSMAD2/3. Longitudinal sectioning of individual penetrating CAA‐laden arterioles showed amyloid deposits along the entire vessel wall, but an absence of calcification along the leptomeninges and the superficial cortical layers. Mid‐way through the cortex, the gradual transition from non‐calcified to calcified vessel wall was accompanied by the occurrence of OPN and pSMAD2/3 granules showing the same gradual transition (Figure 3). Within individual patients, CAA‐laden vessels with different calcification load could be identified within one cortical region (Figure 4). Although calcified vessels were always OPN and pSMAD2/3 positive, the reverse is not the case: some OPN and pSMAD2/3 staining was detected in non‐calcified angiopathic vessels as well.

OPN also labeled the nuclei and cytoplasm of some glial cells and neurons. Notably, perivascular cells with a strong cytoplasmic OPN staining were detected in the proximity of vessels undergoing calcification (Supporting Information Figure S2). In the parenchyma, pSMAD2/3 labeling was predominantly located in the nuclei of neurons, with little or no cytoplasmic staining, as previously described 9.

#### Characterization of the layers in the calcified vessels

Studies in peripheral vessels show that diverse mechanisms may trigger calcification, with different vascular components or cell types involved, such as the vascular smooth muscle cells (VSMCs), the endothelium or the extracellular matrix. Therefore, we investigated the different layers (luminal, medial and abluminal) of the calcified vessels in more detail.

**Figure 5:**
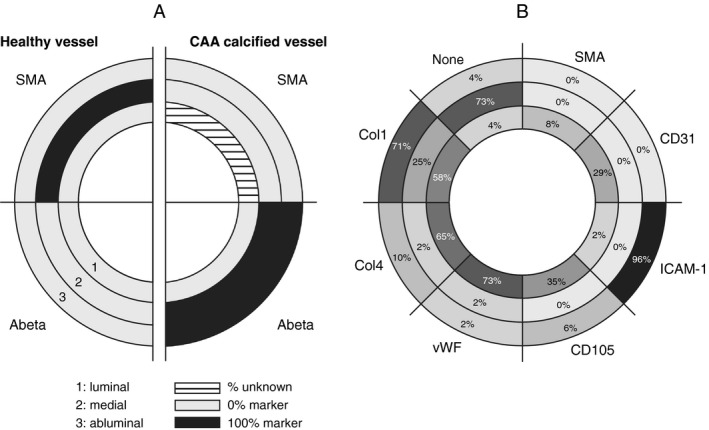
Characterization of calcified vessels. **A**. Representation of Aβ and SMA labeling in the luminal, medial and abluminal vascular tunics in healthy and calcified CAA vessels in HCHWA‐D. Luminal = endothelium; medial = VSMCs; abluminal = external basement membrane and astrocytes end foot. **B**. Representation of the proportion of each staining occurrence (or absence of) per vessel tunics. Most of the vessel tunics scored for multiple markers.

In D‐CAA, the VSMCs have degenerated, as shown by the absence of SMA in the medial layer (Figure 5A). However, the endothelial layer is thought to remain intact 31. Nevertheless, a recent study assessing endothelial dysfunction in CAA showed a loss of CD31 in AD with CAA vessels compared to AD without CAA 21. As endothelial dysfunction may trigger inflammation and is closely linked with vascular pathology, including peripheral vascular calcification, we investigated normal (CD31, CD105, vWF) and activated (ICAM‐1) endothelial markers.

Lumen were most frequently labeled for vWF (73% of calcified CAA vessels; Figure 5B). Classical endothelial labeling CD105 and CD31 remained positive in only 35% and 29% of the calcified CAA vessels, whereas luminal SMA staining was detected in 8% of the quantified vessels. An absence of luminal immunostaining was scored in 4% of the calcified vessels. The endothelial inflammation immunomarker ICAM‐1 was present in 21% of the calcified lumens, whereas ICAM‐1 on astrocyte end foot was the major abluminal staining (96% of the scored calcified vessels; Figure 5B).

ECM remodeling of the basement membrane is known to occur in CAA, initially at the abluminal side. As Col1 is a known modulator of calcification, we investigated the presence of normal (Col 4) and fibrotic (Col 1) collagen. Col1 staining was more frequent than Col4 staining in abluminal (71% vs. 10%) and medial layers (25% vs. 2%) but not in luminal layers (58% vs. 65%; Figure 5B).

In accordance with endothelial dysfunction and ECM remodeling, calcified vessels presented a reduction in classical luminal immunomarkers and an intensification in medial and abluminal fibrotic Col1. The above changes in the different layers were also observed in non‐calcified CAA vessels but much less frequent.

### Vascular accumulation of Col1, OPN and pSMAD2/3 immunomarkers is related to disease severity

We observed that the majority of CAA vessels and capCAA (Aβ‐laden capillaries) in D‐CAA were Col1 positive; anatomically normal vessels were Col1 negative. An illustration of the topographical association between Col1 and Aβ in the angiopathic vessel wall is presented in Supporting Information Figure S3 for two cases with different CAA and capCAA severity. CAA and CapCAA vessels were mostly OPN positive, but also frequently pSMAD2/3 positive.

**Figure 6:**
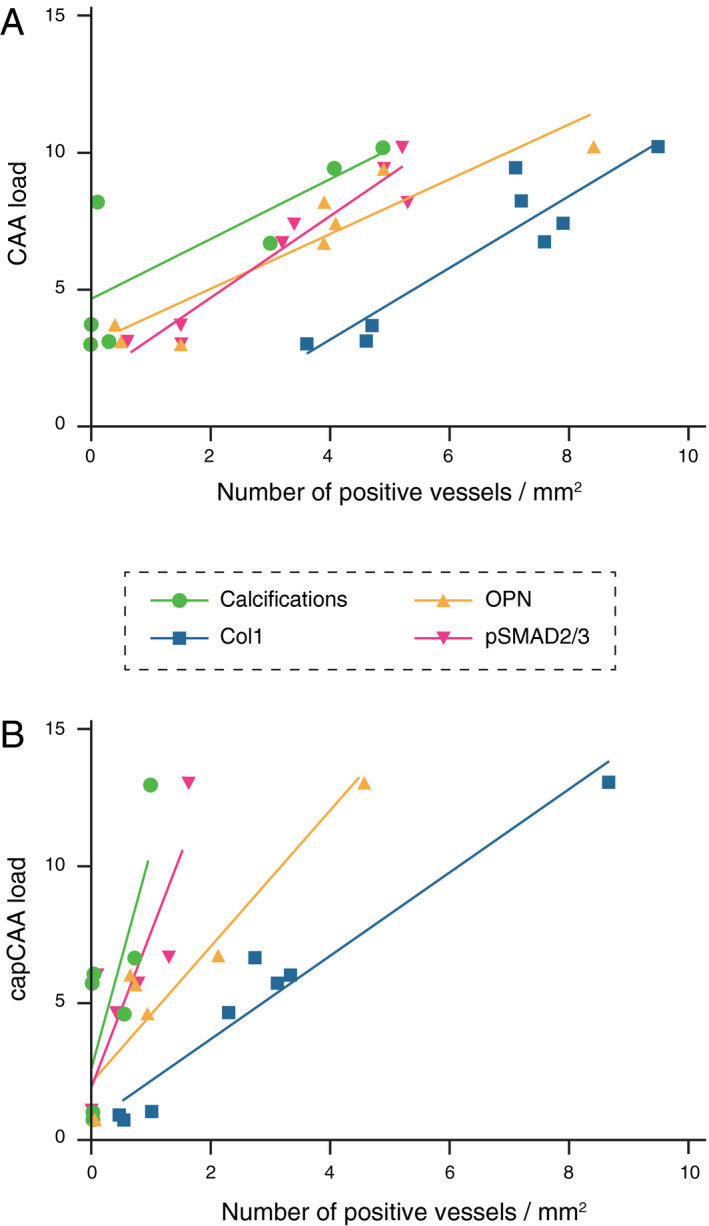
Graphical representation of the quantified OPN, pSMAD2/3 and Col1 immunomarkers and calcified vessels per patient plotted against the CAA load. (**A**) in medium to large vessels and (**B**) in capillaries. Highest correlation coefficient were obtained in A) between the CAA load and the pSMAD2/3 or OPN load (respectively Pearson *r* = 0.96, *P* = 0.0001 and *r* = 0.93, *P* = 0.0009). Details are given in Supporting information Figure S4.

Apart from a topographical association, we also investigated the accumulation of OPN, pSMAD2/3 and Col1 in the vessel wall in relation to disease severity as measured by CAA or capCAA load. Positive significant correlations between all immunomarkers studied and disease severity were found (Figure 6 and Supporting Information Figure S4). Independently of the vessel size, the correlation of CAA/capCAA load with OPN, pSMAD2/3 and Col1 load was most significant.

## Discussion

This study investigated OPN and Col1 immunomarkers thought to promote calcification together with Aβ and pSMAD2/3 known immunomarkers associated with CAA severity, and proposes a mechanism of vascular calcification in D‐CAA.

### Calcifications are a feature of late stage CAA and capCAA pathology

We found a significant correlation of the occurrence of vascular calcification with CAA severity, in line with a previous study on CAA‐associated microvasculopathies (including calcifications) which also showed a correlation with CAA load 27.

Despite the spatial correlation between CAA load and vascular calcification, to date there is no proof for causality between the presence of the Aβ peptide per se, and calcification of the vessel wall 28, 33. This is further supported by our previous finding where we observed calcification of non‐CAA vessels in a sCAA case 5. Furthermore, we observed in the present and in previous studies very heterogeneous patterns of calcification in areas with comparable CAA load, even within the same patient. This suggests that vascular calcifications are most likely a secondary complication of the CAA pathology, but not directly caused by the deposition of amyloid.

As described in prior studies, capCAA is detected only in severely affected D‐CAA patients and increases with CAA load 19, 27. Calcifications of capCAA occurred mostly in subjects with the highest calcified vessel load and in proximity to other calcified vessels. This might either indicate a spreading of the calcification over the entire vascular tree, or it could be the result from surrounding parenchymal changes. Given the preferential occurrence of calcification in specific cortical lamina 5, local changes in the surrounding parenchyma are more plausible.

### Col1, OPN and pSMAD2/3 accumulation might precede vessel wall calcification

To investigate the pathomechanism of vascular calcification in CAA, we investigated several potential modulators. pSMAD2/3 is a direct TGFβ1 downstream signaling factor which is known to modulate peripheral vascular calcification. OPN is a matrix protein induced by TGFβ, also leading to peripheral vessel calcification and a central nervous system constituent of the ECM. Lastly, we stained for Col1, a pro‐fibrotic gene induced by TGFβ1 signaling and an OPN binder, involved in ECM remodeling.

In Figure 6, the order of progression for each immunomarker is visualized as a function of increasing CAA severity. Interestingly, within the same area the number of vessels labeled by Col1 was often higher than the number of vessels stained for Aβ (Supporting Information Figure S4B), indicating that fibrosis of the vessel wall might precede the amyloid deposition. Alternatively, as the arterial system was not distinguished from the venous system, the fibrosis of veins and venules (usually spared from amyloid deposition) might contribute to the number of Col1‐positive vessels. Both scenarios involve an early stage fibrosis of the vessel wall because of ECM remodeling regulated by TGFβ as proposed in our previous work 9, 10.

### TGFβ and OPN changes in calcified vessels

We regularly observed OPN and pSMAD2/3 accumulation in non‐calcified angiopathic vessels, suggesting an ongoing calcification process with OPN and TGFβ as inducing factors of calcification. Literature indeed support this notion, for example, in neuron‐specific TGFβ1‐overexpressing transgenic mice, TGFβ overexpression led to induction of calcification genes a microarray analysis of genes in the cortex of these mice identified upregulation of a group of genes involved in calcium homeostasis, tissue calcification and vascular calcification, including OPN 37. Furthermore Shi *et al* demonstrated that pSMAD3 can bind directly to the OPN promoter, activating OPN transcription 34. Also, SMAD3 overexpression in a mouse osteoblastic cell line was found to promote calcification via Col1 upregulation 35.

### Calcification hypothesis in D‐CAA

Many mechanisms have been proposed for vascular calcification in peripheral vascular diseases. Cortical calcification in this paper resembles concentric medial artery calcification described in diabetes and chronic renal disease 16 and are different from intimal calcification as seen in atherosclerosis (eccentric; lumen deforming; 3). In medial calcification, the proposed mechanisms are usually VSMC‐mediated, with vascular OPN production 2.

However, in our study, VSMCs are likely not responsible for the medial calcification. Firstly, VSMCs are not present in calcified, nor in pre‐calcified OPN‐positive vessels (5 and this study) because CAA‐positive vessels usually do not have any VSMCs remaining. We also speculate that the increased OPN in D‐CAA may not have a vascular origin at all, be it from SMC or other cell types. This is based on our observation that there was local heterogeneity of vascular calcification, even within the same patient. We hypothesize that this variability may be as a result of tissue responses to local events such as microbleeds, micro‐infarcts and hypoxia that are likely to occur in these patients. In this context, OPN is known to be upregulated and neuroprotective after ischemic events, such as stroke 25. Local cortical injuries induce OPN in parenchymal cells as demonstrated in animal models of blood brain barrier damage in the context of ischemic 12 or hemorrhagic injuries 7. Furthermore, OPN is an immunomodulatory cytokine, upregulated in reaction to toxic amyloid species 30. Lastly, it was demonstrated *in vitro*, that OPN could induce the calcification of Col1 fibers in the context of bone formation 32. In the current study the co‐localization of OPN and Col1 in calcified vessels suggests a functional link between vascular OPN likely trapped into Col1 fibers. Testing these hypotheses should be done in future studies using animal or cell culture models.

#### Generalisation of the findings and clinical relevance

Although the proposed mechanisms cannot automatically be generalized to all types of vascular calcifications, it is concordant with previous findings in AD cases where OPN was associated with basal ganglia vascular calcifications 8 and where vascular fibrosis (thickening of the vessel wall) was observed in hippocampal calcifications 42. Although the presence of vascular Aβ in the above mentioned studies was not studied, it suggests an Aβ‐independent mechanism as we propose in the present manuscript, because basal ganglia and hippocampal vessels are relatively exempt of CAA 36.

At this point we do not have sufficient data to draw any conclusions about the clinical relevance of vascular calcification in D‐CAA. We described above that OPN also is known to have neuroprotective properties, and its induction may have a positive effect on the disease course of D‐CAA patients. Whether vascular calcification is an innocent byproduct of this neuroprotective response, or will have negative consequences for future hemorrhagic events in D‐CAA remains to be investigated.

## Conflicts of interest

The authors declare they have no conflicts of interest.

## Ethical approval

All procedures performed in this study involving human participants were in accordance with the ethical standards of the institutional and/or national research committee and with the 1964 Helsinki declaration and its later amendments or comparable ethical standards.

## Informed consent

Informed consent was obtained from all individual participants included in the study.

## Supporting information


**Figure S1.** Example of immunolabelings in a calcified vessel (black on von Kossa or purple with hematoxylin counterstain) identified on serial sections (vessel‐within‐vessel configuration, H14 patient). This calcified vessel scored positively (brown staining) for all immunomarkers (co‐occurrence of seven stainings, single finding). Scale bar 50 μm.
**Figure S2.** Perivascular cells with a strong OPN staining (arrowhead) were detected at proximity of vessels undergoing calcification (arrow). A stronger pSMAD2/3 accumulation is present on the side of the calcification as well (arrow). H2 patient, consecutive slides, scale bar 50 μm.
**Figure S3.** Example of graded area showing the strong correlation between the CAA/capCAA staining and the specific vessel wall Col1 staining in two patients with (A) low capCAA (patient H5) and (B) high capCAA load (patient H9). Scale bar 200 μm.
**Figure S4.** (A) Correlation matrix between all immunomarkers load investigated. Only fully calcified vessels were quantified. Markers load were following a normal distribution (D’Agostino & Pearson normality test; P < 0.05) with the exception of OPN and Col1 load in capillaries. (B) Load in quantified vessel immunomarkers per patient (ranked on increasing CAA load from left to right) in (1) larger vessels (arterioles and veins) and in (2) smallest size vessels (capillaries and venules). Col1 load indicate the total number of fibrotic vessel and might include veins and venules.

**Table S1**. Antibody list and overview of immunohistochemistry protocols.
**Table S2.** Overview of the serial sectioning with the order of staining (n is the first stained slide of the serie; n+x where x indicates the number of 5 μm slides consecutive to n).
