# Supplementary material for: Osteopontin and phospho‐SMAD2/3 are associated with calcification of vessels in D‐CAA, an hereditary cerebral amyloid angiopathy
Source: Brain Pathol. 2019 Apr 4;29(6):793–802. doi: 10.1111/bpa.12721 (PMC6850614; doi:10.1111/bpa.12721)
Supplement: Supplementary file 1 — Figure S1. Example of immunolabelings in a calcified vessel (black on von Kossa or purple with hematoxylin counterstain) identified on serial sections (vessel‐within‐vessel configuration, H14 patient). This calcified vessel scored positively (brown staining) for all immunomarkers (co‐occurrence of seven stainings, single finding). Scale bar 50 μm. Figure S2. Perivascular cells with a strong OPN staining (arrowhead) were detected at proximity of vessels undergoing calcification (arrow). A stronger pSMAD2/3 accumulation is present on the side of the calcification as well (arrow). H2 patient, consecutive slides, scale bar 50 μm. Figure S3. Example of graded area showing the strong correlation between the CAA/capCAA staining and the specific vessel wall Col1 staining in two patients with (A) low capCAA (patient H5) and (B) high capCAA load (patient H9). Scale bar 200 μm. Figure S4. (A) Correlation matrix between all immunomarkers load investigated. Only fully calcified vessels were quantified. Markers load were following a normal distribution (D’Agostino & Pearson normality test; P < 0.05) with the exception of OPN and Col1 load in capillaries. (B) Load in quantified vessel immunomarkers per patient (ranked on increasing CAA load from left to right) in (1) larger vessels (arterioles and veins) and in (2) smallest size vessels (capillaries and venules). Col1 load indicate the total number of fibrotic vessel and might include veins and venules. [file BPA-29-793-s001.pdf]

Supplementary Figures

Article title: Osteopontin and pSMAD2/3 are associated with calcification of vessels in hereditary cerebral amyloid angiopathy

Journal: Brain Pathology (2018)

Authors and affiliations: Laure Grand Moursel[1,2], Linda M. van der Graaf[1,2], Marjolein Bulk[2], Willeke M.C. van Roon-Mom[1], Louise van der Weerd[1,2]

[1]Department of Human Genetics, Leiden University Medical Center
[2]Department of Radiology, Leiden University Medical Center

*__Corresponding author__
Leiden University Medical Center, Einthovenweg 20, 2333 ZC Leiden, The Netherlands
Phone: +31-71-526 9603 E-mail: L.Grand_Moursel@lumc.nl

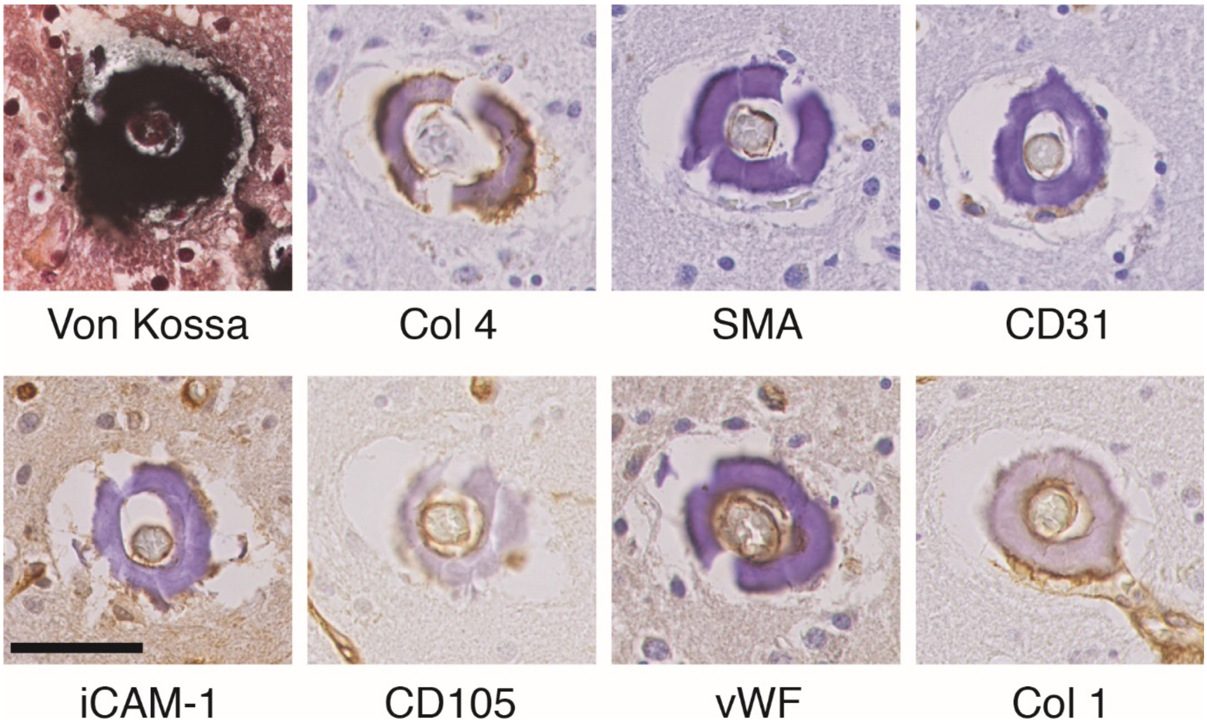

Supplementary Figure S1: Example of immunolabelings in a calcified vessel (black on von Kossa or purple with hematoxylin counterstain) identified on serial sections (vessel-within-vessel configuration, H14 patient). This calcified vessel scored positively (brown staining) for all immunomarkers (co-occurrence of 7 stainings, single finding). Scale bar 50µm.

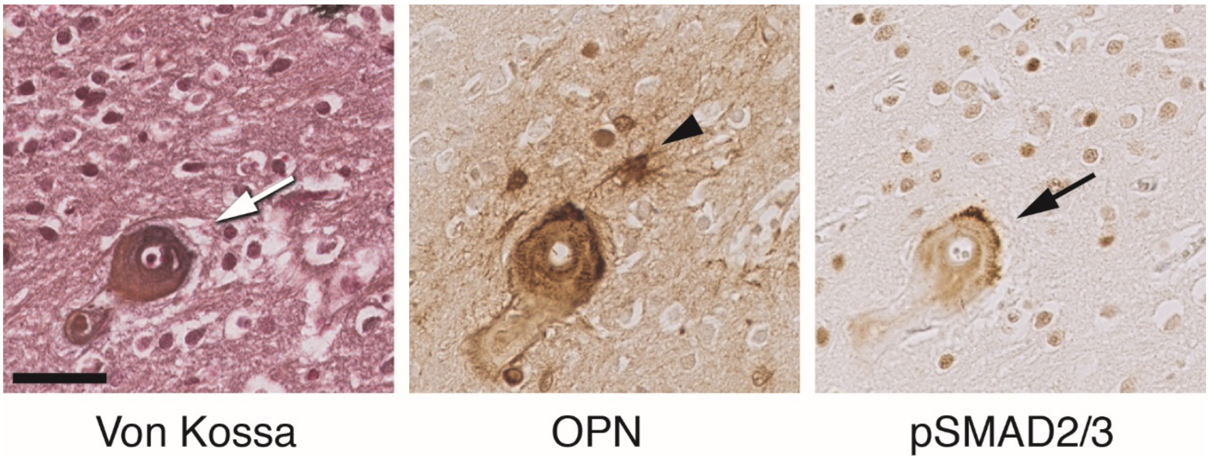

Supplementary Figure S2: Perivascular cells with a strong OPN staining (arrowhead) were detected at proximity of vessels undergoing calcification (arrow). A stronger pSMAD2/3 accumulation is present on the side of the calcification as well (arrow). H2 patient, consecutive slides, scale bar 50µm.

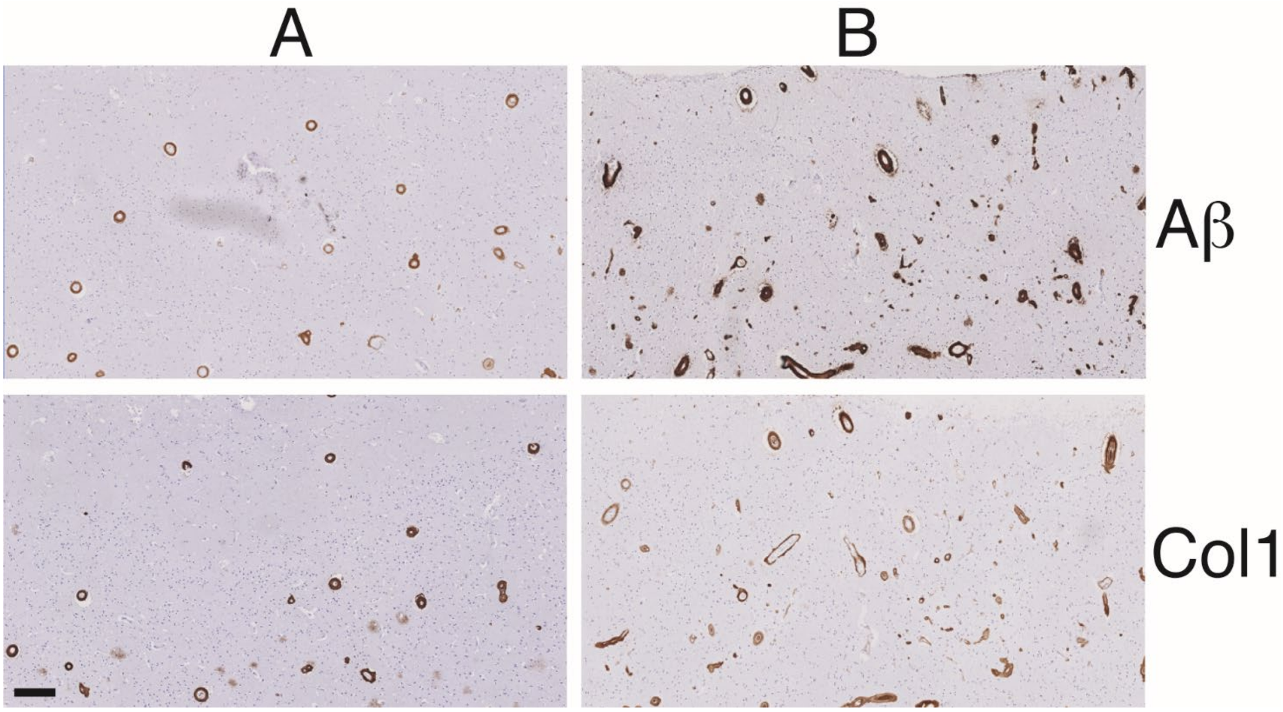

Supplementary Figure S3: Example of graded area showing the strong correlation between the CAA/capCAA staining and the specific vessel wall col1 staining in two patients with (A) low capCAA (patient H5) and (B) high capCAA load (patient H9). Scale bar 200µm.

Medium to large vessels

Capillaries

A1 *Pearson's r*

	Calcifications	Col1	OPN	pSMAD2/3
Col1	0.75			
OPN	0.84	0.92		
pSMAD2/3	0.63	0.84	0.86	
CAA	0.77	0.92	0.93	0.96

$p > 0,05$
$p \leq 0,05$
$p \leq 0,01$
$p \leq 0,001$

A2 *Pearson's r*

	Calcifications	pSMAD2/3
pSMAD2/3	0.85	
OPN	0.78	0.88

Spearman's r

	Calcifications	Col1	OPN
Col1	0.53		
OPN	0.85	0.71	
pSMAD2/3		0.81	0.95
CAA		0.90	0.81

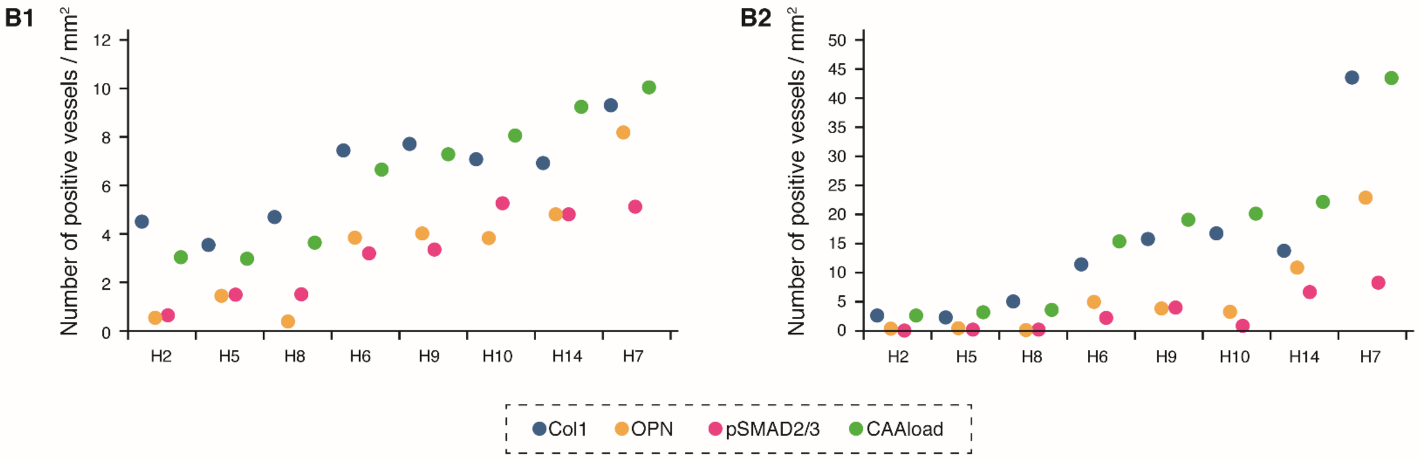

Supplementary Figure S4: (A) Correlation matrix between all immunomarkers load investigated. Only fully calcified vessels were quantified. Markers load were following a normal distribution (D'Agostino & Pearson normality test; $p<0.05$) with the exception of OPN and Col1 load in capillaries. (B) Load in quantified vessel immunomarkers per patient (ranked on increasing CAA load from left to right) in (1) larger vessels (arterioles and veins) and in (2) smallest size vessels (capillaries and venules). Col1 load indicate the total number of fibrotic vessel and might include veins and venules.
