# Supplementary material for: Osteopontin and phospho‐SMAD2/3 are associated with calcification of vessels in D‐CAA, an hereditary cerebral amyloid angiopathy
Source: Brain Pathol. 2019 Apr 4;29(6):793–802. doi: 10.1111/bpa.12721 (PMC6850614; doi:10.1111/bpa.12721)
Supplement: Supplementary file 2 — Table S1. Antibody list and overview of immunohistochemistry protocols. Table S2. Overview of the serial sectioning with the order of staining (n is the first stained slide of the serie; n+x where x indicates the number of 5 μm slides consecutive to n). [file BPA-29-793-s002.pdf]

Supplementary Tables

Article title: Osteopontin and pSMAD2/3 are associated with calcification of vessels in hereditary cerebral amyloid angiopathy

Journal: Brain Pathology (2018)

Authors and affiliations: Laure Grand Moursel[1,2], Linda M. van der Graaf[1,2], Marjolein Bulk[2], Willeke M.C. van Roon-Mom[1], Louise van der Weerd[1,2]

[1]Department of Human Genetics, Leiden University Medical Center
[2]Department of Radiology, Leiden University Medical Center

***Corresponding author**
Leiden University Medical Center, Einthovenweg 20, 2333 ZC Leiden, The Netherlands
Phone: +31-71-526 9603 E-mail: L.Grand_Moursel@lumc.nl

Supplementary Table 1 Antibody list and overview of immunohistochemistry protocols

Name	Host	Dilution	Company & Type	Reference	AR[a]	Enhancement; DAB reagent, supplier
pSMAD2/3	Rb (pc)	1:500	Cell Signaling	3101S	40 min boiling[#]	No; SK-4100, Vector lab
Osteopontin	Gt (pc)	1:1000	R&D systems	AF1433	20 min boiling, 30 min cooling down	No; SK-4100, Vector lab
Abeta	Ms (mc)	1:20	Dakocytomation	M0872	Trypsin/Formic acid treatment	ABC (PK-6100, Vector lab)
SMA	Ms (mc)	1:700	Dakocytomation	M0851	No	ABC (PK-6100, Vector lab)
Collagen IV	Rb (pc)	1:400	Abcam	ab6586	20 min Proteinase K, 10 min cooling down	No; SK-4100, Vector lab
CD31	Ms (mc)	1:150	Dakocytomation	M0823	20 min boiling, 30 min cooling down	ABC (PK-6100, Vector lab)
iCAM-1	Ms (mc)	1:200	Santa Cruz	sc-8439	20 min boiling, 30 min cooling down	No; SK-4100, Vector lab
Endoglin	Gt (pc)	1:200	R&D systems	AF1097	20 min boiling, 30 min cooling down	No; SK-4100, Vector lab
vWF	Gt (pc)	1:500	Kordia		No	ABC (PK-6100, Vector lab)
Collagen I	Gt (pc)	1:300	Southern Biotechnology	1310-01	20 min Proteinase K, 10 min cooling down	No; SK-4100, Vector lab

AR Antigen retrieval, Rb Rabbit, Ms Mouse, Gt Goat, pc polyclonal, mc monoclonal, min minutes, vWF von Willebrand Factor

[a] all boiling steps in acidic pH 6 solution (H-3300, Vector labs)
[#] in pressure cooker as described in Grand Moursel *et al,* 2017 (16)

Supplementary Table 2 Overview of the serial sectioning with the order of staining (n is the first stained slide of the serie; n+x where x indicates the number of 5 µm slides consecutive to n)

pSMAD2/3	Osteopontin	Von Kossa	Abeta	Collagen-IV	SMA	CD31	iCAM-1	Endoglin	HE	vWF	Collagen-I
n	n+1	n+2	n+3	n+4	n+5	n+6	n+7	n+8	n+10	n+11	n+12
